# On the Evaluation of the NB-IoT Random Access Procedure in Monitoring Infrastructures

**DOI:** 10.3390/s19143237

**Published:** 2019-07-23

**Authors:** Sergio Martiradonna, Giuseppe Piro, Gennaro Boggia

**Affiliations:** 1 Department of Electrical and Information Engineering—Politecnico di Bari, 70126 Bari, Italy; 2 CNIT—Consorzio Nazionale Interuniversitario per le Telecomunicazioni, 43124 Parma, Italy

**Keywords:** monitoring system, sensor network, NB-IoT, random access channel, simulation tool, analytical model

## Abstract

NarrowBand IoT (NB-IoT) is emerging as a promising communication technology offering a reliable wireless connection to a large number of devices employed in pervasive monitoring scenarios, such as Smart City, Precision Agriculture, and Industry 4.0. Since most of the NB-IoT transmissions occur in the uplink, the random access channel (that is the primary interface between devices and the base station) may usually become the main bottleneck of the entire system. For this reason, analytical models and simulation tools able to investigate its behavior in different scenarios are of the utmost importance for driving current and future research activities. Unfortunately, scientific literature partially addresses the current open issues by means of simplified and, in many cases, not standard-compliant approaches. To provide a significant step forward in this direction, the contribution of this paper is three-folded. First, it presents a flexible, open-source, and 3GPP-compliant implementation of the NB-IoT random access procedure. Second, it formulates an analytical model capturing both collision and success probabilities associated with the aforementioned procedure. Third, it presents the cross-validation of both the analytical model and the simulation tool, by taking into account reference applications scenarios of sensor networks enabling periodic reporting in monitoring infrastructures. Obtained results prove the remarkable accuracy, demonstrating a well-calibrated instrument, which will be also useful for future research activities.

## 1. Introduction

The Internet of Things (IoT) phenomenon is broadening at an astoundingly fast rate [1], which promoted the birth of several novel services in different application domains, including, but not limited to, Industry 4.0 [2], Smart Cities [3], Intelligent Transportation Systems [4], Precision Agriculture [5], healthcare [6] and environmental monitoring [7]. Hence, an increasing number of constrained smart devices are currently joining the worldwide Internet, asking for suitable wireless communication technology, capable of offering both extremely low power consumption and support for densely populated deployments. In general, these low-powered devices individually require low data transfer rates as well, even though a densely populated Machine Type Communication (MTC) deployment might become incredibly bandwidth-hungry. At the same time, devices generally require extremely low power, remaining idle for prolonged periods. Furthermore, reasonably numerous IoT applications involve the reporting of various events and data streaming to a central server in an efficient and robust way (e.g., video surveillance and emergency health services). Besides, either the physical location of devices in such scenarios may not be reached by fixed networks (e.g., basements) or the apparatuses have to be arbitrarily deployed or moved anywhere [8]. Low Power Wide Area networks (LPWANs) are an innovative communication pattern addressing the aforementioned requirements of emerging IoT applications [9]. Essentially, they complement legacy cellular and short-range wireless technologies, offering exclusive features for low complexity devices. At the time of this writing, there are a number of LPWANs, each employing different techniques to meet the Machine-to-Machine (M2M) requirements. In the unlicensed spectrums, LoRa [10] and SigFox [11] are the most common. However, the licensed spectrum is widely known to supply a higher degree of reliability and Quality of Service. Among the licensed LPWANs, NarrowBand IoT (NB-IoT) has been recognized as a promising and effective technology offering wireless connectivity to smart devices. Standardized by the Third Generation Partnership Project (3GPP) starting from Release 13 [12,13], it natively supports the transmission of marginal amounts of data, while requiring low-energy consumption and limited bandwidth usage [14]. Moreover, the adoption of NB-IoT is able to satisfy most of the requirements of all the possible application scenarios, by dynamically adapting to different use cases. Therefore, the application based on the potentially enormous amount and variety of data generated by monitoring sensors is of particular interest. Such monitoring systems provide new services to citizens, companies, and public administrations as well.

In a typical NB-IoT monitoring system, uplink transmissions prevail among the rest of exchanged messages. Here, the random access procedure is the primary relation established between devices and the base station. Built on top of a contention-based mechanism, it brings to performance degradation when a multitude of simultaneous transmissions takes place [15]. In fact, in high traffic conditions, only few devices can successfully complete the procedure. Consequently, analytical models and system-level simulation tools able to investigate the behavior of the aforementioned random access procedure are extremely important for driving current research activities related to the adoption of the NB-IoT technology in smart monitoring infrastructures.

Nevertheless, at the time of this writing, scientific literature only provides splintered answers to these challenging open issues. For instance, analytical models for estimating the random access success probability are presented in [16,17,18,19,20,21,22], but they either do not fit the 3GPP guidelines or overlook several aspects (see Section 2.4 for further details). In addition, to make matters worse, available simulation tools do not completely support the random access procedure evaluation [23,24] and the NB-IoT open-source simulation presented in [25] does not offer a satisfactory implementation of the procedure itself.

Based on these premises, this contribution provides a significant step forward in the current state of the art, by overcoming the evident limitations characterizing the scientific literature and presenting key instruments for the evaluation of the NB-IoT random access procedure. For the first time, to the best of the authors’ knowledge, the NB-IoT random access procedure is jointly investigated through analytical models and system-level simulations, paving the way for future standard improvements. Specifically, the contribution of this paper is three-fold.

1. Starting from the platform presented in [25], it puts forward an open-source and 3GPP-compliant implementation of the NB-IoT random access procedure, which handles different coverage classes, global and local transmission attempts counters, backoff times, and transitions from a class to another, as well as other key simulations.
2. It formulates an analytical model describing both collision and success probabilities, given the number of users willing to perform the random access procedure.
3. It deeply cross-validates the analytical model and the simulation tool, by considering realistic NB-IoT deployments enabling periodic reporting in monitoring infrastructures, with different resource configurations.

The rest of this work is organized as follows. Section 2 illustrates the NB-IoT technology, describing in details the Random Access Procedure, and the currently available analytical models and network simulators, as well as several real NB-IoT monitoring systems. Section 3 presents the implemented simulation tool and the conceived analytical model. Section 4 discusses the results obtained through the cross-validation process. Section 5 concludes the paper and draws future research activities.

## 2. State of the Art

### 2.1. NarrowBand—Internet of Things

NB-IoT is a cellular radio access technology that requires a smaller bandwidth than Long Term Evolution (LTE), that is, 180 kHz for both downlink and uplink [13]. A single carrier can be configured according to the chosen operation mode, whereas multiple carriers can also be used to supply a higher bandwidth. The three possible operation modes include:

- **Stand-Alone** operation: An operator can replace one GSM carrier of 200 kHz with NB-IoT, leaving a guard interval of 10 kHz on both sides of the spectrum.
- **In-Band** operation: One or more NB-IoT carriers are deployed inside a larger LTE channel.
- **Guard-Band** operation: One or more carriers are allocated within the guard-band of LTE bandwidth to NB-IoT.

All three deployment scenarios are transparent to non-NB-IoT devices. Consequently, LTE devices that do not implement NB-IoT functionality simply do not see the NB-IoT channel inside the main LTE bandwidth or in the guard band. At the same time, legacy GSM devices will not see an NB-IoT carrier if used alongside 180 kHz GSM carriers. Such devices will only see noise where NB-IoT is active [26].

At the physical layer, NB-IoT fully inherits from LTE in the downlink. The transmission scheme is based on conventional Orthogonal Frequency Division Multiplexing (OFDM) using normal Cyclic Prefix (CP). An end user operates in the downlink using seven consecutive symbols on 12 subcarriers (also referred to as tones) grouped into a Resource Block (RB), with a subcarrier spacing of 15 kHz [13]. The duration of a slot, subframe, and frame is analogous to LTE, as well. Therefore, in a single NB-IoT carrier, there is only one RB and only one user can receive data at a time using all 12 subcarriers for each subframe. As regards modulation, only Quadrature Phase-Shift Keying (QPSK) is supported.

Instead, the uplink supports not only the standard LTE subcarrier spacing of 15 kHz but also a subcarrier spacing of 3.75 kHz. The elementary NB-IoT radio resource, termed Resource Unit (RU), is the smallest unit to map a transport block [27]. It is assigned to a single user only. Unlike the well-known resource block of LTE, an RU is dynamically defined as shown in  Table 1 [28].

Specifically, the uplink supports two different configurations: *Single-Tone* and *Multi-Tone*. *Single-Tone* uses either 3.75 or 15 kHz subcarrier spacing and each subcarrier represents an RU. Consequently, a 180 kHz carrier is divided into either 48 or 12 RUs, respectively, thus resulting in two different RU length values: 32 and 8 ms. In the case of *Multi-Tone* configuration, the subcarrier spacing is set to 15 kHz only. Nevertheless, 3, 6 or 12 adjacent subcarriers may shape a single RU. Again, depending on the number of tones per RU, its length changes accordingly (as illustrated in  Table 1).

The modulation schemes available in the uplink are restricted to Binary Phase-Shift Keying (BPSK) and QPSK.

One of the main objectives defined in NB-IoT study item description [29] is to achieve 20 dB coverage extension compared with legacy General Packet Radio Service (GPRS) while limiting the device maximum transmit power to 23 dBm (200 mW), which is a factor of ten lower than the maximum output power of GPRS devices. Coverage extension is achieved by increasing the number of repetitions at the expense of higher data rates. Moreover, NB-IoT supports up to three coverage classes, namely *Normal*, *Extended*, and *Extreme*, to serve devices experiencing different ranges of path loss. The *Extreme* class corresponds to incredibly low received power levels. The *Normal* class refers to considerably higher received power levels. Specifically, the end device is considered in a specific enhanced coverage level if its measured Reference Signal Received Power (RSRP) is less than the RSRP threshold configured for that enhanced coverage class. According to the 3GPP specification [30], the number of coverage classes is configurable by system information. Let *n* be the number of available coverage classes so that 1≤n≤3. Different coverage classes correspond to operation with different modulation orders, coding rates, repetition factors, and subcarrier spacings, in order to match the data rate for each user to its available link budget. This allows devices having good coverage to operate at higher data rates and with lower latency than devices that have poor coverage. Therefore, the system is designed to meet the throughput and latency requirements for devices in *Extreme* coverage, while devices in *Normal* or *Extended* coverage achieve improved performance.

NB-IoT generally takes advantage of the existing LTE physical channels, revised properly to fit into the narrower bandwidth [31]. In the downlink direction, there are three channels:

- Narrowband Physical Downlink Control Channel (NPDCCH) carries scheduling assignments, as well as Hybrid Automatic Repeat reQuest (HARQ) acknowledgments, paging indication, and system information update. It is considered as the core element of the scheduling procedure as it carries Downlink Control Indicators (DCIs), which contain uplink scheduling grants, downlink scheduling assignments, and the type of modulation.
- Narrowband Physical Downlink Shared Channel (NPDSCH) carries data from the higher layers as well as paging message, system information messages, and the Random Access Response (RAR) message. It is scheduled by the NPDCCH but is transmitted after a certain time delay (indicated in the NPDCCH too) to allow the low-complexity NB-IoT devices enough time to decode the NPDCCH.
- Narrowband Physical Broadcast Channel (NPBCH) is transmitted in the first subframe and carries the Master Information Block - NarrowBand (MIB-NB) over eight consecutive radio frames, repeated 8 times to cope with extreme coverage conditions; the MIB-NB content remains, therefore, unchanged for 640 ms.

On the other hand, for the uplink direction, there are two different channels, specifically:

- Narrowband Physical Uplink Shared Channel (NPUSCH) has two formats. Format 1 is used for carrying uplink data and uses the same LTE error correction code. Format 2 is used for signaling HARQ acknowledgment for NPDSCH and uses a repetition code for error correction.
- Narrowband Physical Random Access Channel (NPRACH) enables the random access procedure. Thus, it is described in details in the following section.

**Figure 1:**
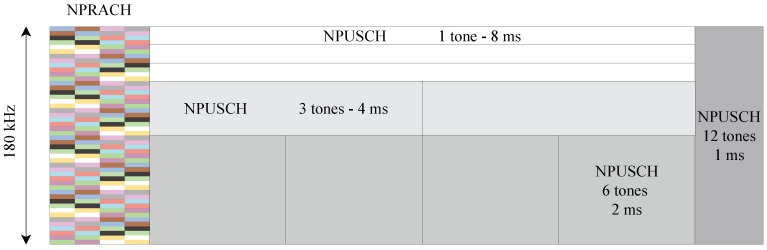
Time–frequency structure of NB-IoT uplink channels.

Figure 1 shows a representative time–frequency structure of NB-IoT uplink channels when the subcarrier spacing of the NPUSCH is set to 15 kHz.

As regards synchronization signals, Narrowband Primary Synchronization Signal (NPSS) and Narrowband Secondary Synchronization Signal (NSSS) are used by an NB-IoT device to perform cell search, which includes time and frequency synchronization and cell identity detection.

### 2.2. NPRACH and Random Access Procedure

The NPRACH has been completely redesigned [20]. In fact, the traditional LTE PRACH alone occupies a much greater bandwidth than the entire NB-IoT carrier (e.g., 1.05 MHz vs. 180 kHz). Moreover, since the LTE PRACH symbols are based on Zadoff–Chu sequences, the related signal has a non-zero peak-to-average power ratio, that reduces coverage while increasing the energy footprint. Differently, NPRACH only uses the *Single-Tone* configuration and 3.75 kHz subcarrier spacing, hence occupying between 45 kHz and 180 kHz, according to the number of subcarriers.

**Figure 2:**
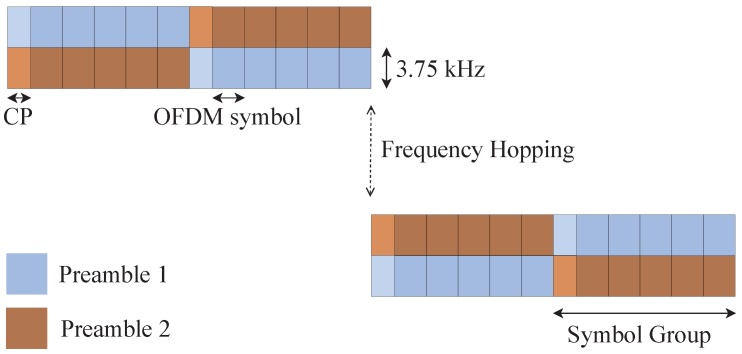
NPRACH preambles.

**Figure 3:**
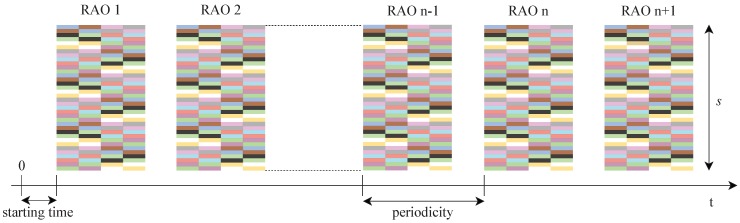
RAOs timing diagram.

The NB-IoT random access preamble is shown in Figure 2. It is mainly characterized by four symbol groups, each of them comprising five OFDM symbols plus the cyclic prefix. In particular, the network configures the preamble format (i.e., the duration of the cyclic prefix), depending on the cell size. A single NPRACH preamble lasts either 5.6 ms or 6.4 ms, when Format 0 or Format 1 is applied, respectively. A pseudo-random frequency hopping algorithm offers as different preambles the number of subcarriers allocated to the NPRACH. Consequently, selecting different subcarriers at the beginning of the transmission ensures hopping schemes which never overlap. In addition, different NPRACH resource configurations can be deployed in a cell, each corresponding to a different coverage class. Each one is described through periodicity, the number of repetitions, starting time, frequency location, and the number of subcarriers, as depicted in Figure 3. Generally, an NPRACH resource is called Random Access Opportunity (RAO).

In contrast, LTE PRACH structure is defined by predefined configuration indexes, which are broadcasted by System Information as well. In the time domain, the LTE PRACH occupies one, two or three subframes (i.e., 1, 2 or 3 ms, respectively) and it is periodically scheduled by the base station, while in the frequency domain it needs exactly six LTE RBs. The PRACH always consists of a set of 64 orthogonal sequences. Typically, the PRACH is scheduled every 5 ms and 54 preambles are dedicated to the contention-based access, while the other 10 are reserved for contention-free access.

**Figure 4:**
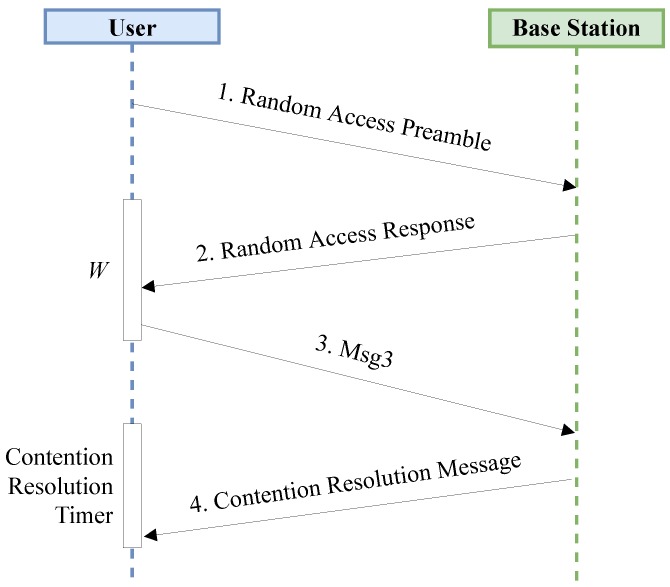
Random access procedure sequence diagram.

On the other hand, NB-IoT random access procedure is per se contention based [30] and entails the exchange of four messages, as depicted in Figure 4:

1. The device finds the first available RAO and then transmits a random preamble, chosen among the available ones. Let *s* be the number of the available subcarriers. Then, the end device starts the RAR window, *W*.
2. Upon preamble reception, the base station transmits an RAR that explicitly instructs the user on which uplink resources have to be utilized for the transmission of the next message. If this message is not received, the user keeps waiting for it until the expiration of *W*.
3. Exploiting the scheduled resources, the user transmits its identity and other important information; this message is also known as Msg3. Subsequently, the terminal starts the Contention Resolution Timer.
4. The base station performs the contention resolution and sends back to the devices the identity of the winning users through the Contention Resolution Message. If this message is not received, users keep waiting for it up to the Contention Resolution Timer expiration.

The procedure fails when either the RAR or the Contention Resolution Message is not correctly received by mobile terminals in the proper windows. Henceforward, a worst-case scenario is assumed. Particularly, overlapping preambles always destructively interfere, therefore the base station never detects them. In other words, if two or more devices send the same preamble in a single RAO, each user fails the procedure.

Each collided user may retransmit a preamble after a backoff time *b*, chosen uniformly and at random within the interval [0,B]. The maximum number of preamble transmission that a single mobile terminal can attempt in a general coverage class *c* is set to $a_{c}$. If a user fails the $a_{c}$th attempt, it considers being in the next higher coverage class, if exists. This behavior is repeated until the maximum number of transmissions that can be tried globally in all classes, i.e., α, is reached.

**Figure 5:**
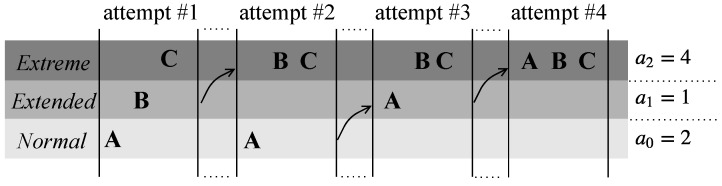
Coverage class hopping of three distinct users during random access procedure with α=4.

As a case in point, Figure 5 reports three different users, i.e., A, B and C, starting the first transmission attempt in the *Normal*, *Extended* and *Extreme* class, respectively, based on the assumption that they always collide with other users. Since $a_{0}=2$, as soon as User A fails the second attempt, it retries the subsequent transmission in the *Extended* coverage class. Here, both A and B can try up to $a_{1}=1$ preamble transmission, hence jumping to the *Extreme* class in the immediately following attempt. On the other hand, User C, whose class is *Extreme*, is forced to persist in its class. Anyway, even though $a_{2}=3$, a total amount of α=4 attempts are foreseen for User C as well.

### 2.3. NB-IoT in Smart Monitoring Infrastructures

NB-IoT appeared as a key technology for offering wireless connectivity to constrained smart devices, while supporting a massive deployment of sensors. By dynamically adapting its features, NB-IoT is able to satisfy most of the requirements of application scenarios distinguished by a high density of terminals, e.g., Smart City, Industry 4.0, and Precision Agriculture. As a matter of fact, several applications based on smart NB-IoT monitoring infrastructures have already been conceived.

Zhang et al. [32] proposed a general information monitoring approach combining NB-IoT and LoRa communication and a solar power supply system. In particular, the LoRa subnode collects sensor information and conducts point-to-point communications with the main node. Then, it processes the received data and sends information to the server through the NB-IoT communication module. Furthermore, the uthors suggested applying their system for grassland and lake information monitoring infrastructures.

The Smart City is the main theme in [33,34,35,36,37,38,39]. Specifically, the study in [33] considers some of the key challenges related to smart cities to study the energy consumption, the latency, and scalability for NB-IoT and eMTC technologies. In addition, it formulates the data transmission delay and the maximum number of devices per cell based on different network configurations, by introducing a network simulator tool based on NS-3. Malarski et al. [34] placed a grid of ultrasonic or infrared sensors at different harbor spots in order to monitor their occupancy. Collected sensor data are then sent to the administration, becoming able to smartly manage spot reservation and release. Based on the measurements taken in a Danish harbor, the authors indeed demonstrated the suitability of the NB-IoT technology as a signal carrier for maritime applications as well, even though water and boat masts partially affect the transmission. A smart parking system is proposed in [35] in order to reduce the deployment cost and improve the user payment experience. In the proposed system, data of sensor nodes installed in parking spaces is transmitted by NB-IoT, whereas charge management, sensor node surveillance, task management, and business intelligence modules are implemented on a cloud server. The system was then actually deployed in two Chinese cities for improving the utilization of existing parking facilities effectively. Chen et al. [36] presented an indoor environment-temperature monitoring system based on NB-IoT sensors and then validated it by means of real experimental results. Pan et al. [37] addressed the issue of urban garbage management by designing a smart garbage bin based on NB-IoT technology. In particular, they employed infrared and odor sensors to provide garbage detection and classification. As a result, the proposed system changes the existing urban sanitation work mode and improves the efficiency of sanitation workers, at the same time promoting the reuse of recyclable resources. Instead, [38] designs an intelligent LED street light control system aiming at saving energy and reducing the maintenance cost and difficulty of the urban street lighting. It consists of a cloud server, a remote monitoring interface, and street light control terminals communicating through NB-IoT. This solution realizes local intelligent control and remote supervision of street lights and, thanks to an energy-saving algorithm, the energy consumption of the street lighting is greatly reduced. Finally, in [39], the authors tried to address the problem of air pollution in a Smart City context. In particular, they developed five types of air pollution detection sensors based on the NB-IoT network in Thailand. Experimental results demonstrate the feasibility of inspecting real-time air quality as a service.

On the other hand, in [40], NB-IoT has been identified as one of the most suitable wireless communication technologies for Smart Agriculture applications because of its key features (low power consumption, long communication range, and relatively easy network implementation). Specifically, NB-IoT sensors collect environmental and soil data, which include ambient temperature, atmospheric humidity, luminance, and soil moisture and temperature, every half an hour and transmit them to the application server. Data are then analyzed by artificial intelligence systems, which impart the corresponding methodologies to actuators to satisfy the specific conditions for crop growths. Similarly, Chang et al. [41] presented a system for monitoring greenhouses of high-cost crop in the suburb. Temperature and humidity data, as well as lighting parameters, are regularly collected from the remote field and then synchronized with servers using NB-IoT. Experimental results further demonstrate the feasibility and stability of data collecting using such a system.

Industry 4.0. was targeted by Rohit et al. [42], who proposed a smart solution for identification, assessment, and tracking of industrial assets. After a comprehensive comparative study between the existing communication technologies, NB-IoT is recognized as a key technology for achieving the requirements of the 4.0 paradigm. In the context of the intelligent fishery, the authors of [43] designed a fully-automatic and intelligent monitoring system for dissolved Oxygen of aquaculture water, in order to reduce the mortality of bred animals. NB-IoT modules, along with optical and polarographic sensors, and controllers are used to maintain the oxygen level of the aquaculture water in the typical range.

As for the Smart Grid, Li et al. [44] demonstrated that NB-IoT work well in four typical communication scenarios, after performing a comprehensive investigation on their requirements. The authors claimed that the needs for field trips of personnel for meter reading, manual outage reporting, and most restoration operations, especially for harsh environments that are difficult for people to access, can be easily eliminated through the employment of NB-IoT.

Zhang et al. [45] formalized an architecture using NB-IoT to connect all intelligent things in smart hospitals and introduce edge computing to deal with the requirement of latency in medical processes. Furthermore, the authors designed an infusion monitoring system based on infrared sensors to monitor the real-time drop rate and remaining drug volume during the intravenous infusion.

Popli et al. [46] delivered a comprehensive study of NB-IoT, extensively elaborating resource allocation and energy efficiency techniques. Moreover, the proposed two novel application specific energy efficient approaches for Smart Agriculture and Smart Health. Specifically, in the first scenario, NB-IoT soil moisture sensors deployed in a farm field send data that are then processed using deep learning to meticulously divide the field into different zones. As for Smart Health scenario, a novel approach for adaptively distributing the transmission power required to monitor the patient is proposed, in order to perform power optimization and greatly improve the lifetime and performance of the monitoring systems.

### 2.4. Current Evaluations of the Random Access Procedure

In a typical NB-IoT monitoring system, uplink transmissions prevail among the rest of exchanged messages. Consequently, the random access procedure represents a key relationship between devices and the base station. However, severe performance degradation occurs in the network when a multitude of simultaneous transmissions takes place since the random access procedure is built on top of a contention-based mechanism. Therefore, analytical models and system-level simulation tools able to investigate the behavior of the aforementioned random access procedure are extremely important for smart monitoring infrastructures. Current scientific literature offers some interesting analytical models for estimating the random access success probability. Nevertheless, depending on both the significant effort to provide a tractable analysis framework and the specific focus of each work, available studies still lack various features. In particular, Sun et al. [16] applied a Markov chain to model retransmission number in a steady-state system with a Poisson arrival process. Instead, the authors of [17] derived the success probability under time correlated interference by considering different outage conditions. Nonetheless, both these systems are considerably distant from 3GPP specifications, hence resulting in inadequacy for veridical performance assessment. In [18], the main 3GPP parameters associated with the random access procedure are investigated to determine how they influence the design of network capacity, the average delay, and normalized flow rate. However, the authors wholly neglected multiple coverage classes. The same happens in [19], where the authors tried to optimally trade repetitions and retransmission values to achieve a target successful probability. Besides, Lin et al. [20] and Hwang et al. [21] focused on the preamble transmission to investigate the performance in terms of detection probability, taking into account either the standard preamble design or a new detection and synchronization algorithm, respectively. However, the random access procedure is not considered as a whole, since only the first message is analyzed. Finally, Harwahyu et al. [22] presented a detailed model for the random access procedure, thereafter employed to perform a joint optimization technique to maximize the access success probability. Nevertheless, it still exposes different flaws. Differently from the standard [47], it only considers RAOs of different classes with the same starting time, hence constraining the total number of subcarriers. Besides, it only considers users requests generated according to the Poisson statistics and it has not been validated through reference tools of recognized importance. In addition, the authors extended a multiband multichannel slotted ALOHA framework, where the collision probability is only approximated through the bins and balls problem.

The discussion above demonstrates clearly that simulation tools could be beneficial for addressing several open issues and driving future research activities. Such a simulation platform would be useful to validate the available analytical frameworks and to conduct performance assessments of entirely new studies. Nonetheless, with respect to recent simulation tools, only preliminary developments of NB-IoT are proposed. Foni et al. [23] presented an ns-3 implementation that is largely incomplete, encompassing solely the signaling protocol. In addition, Miao et al. [24] only proposed a link-level simulator, which is not freely available for the research community. Moreover, the NB-IoT open-source simulation presented in [25] does not offer a complete and standard-compliant implementation of the random access procedure.

It is reasonable to conclude that state-of-the-art frameworks aiming to model contention-based access in NB-IoT systems are extremely important. However, from this review, it is evident that a 3GPP-compliant NB-IoT simulation platform is still missing in the current literature. Consequently, this paper addresses this gap. In particular, the contribution of this paper is three-fold. First, starting from the platform presented in [25], it puts forward an open-source and 3GPP-compliant implementation of the NB-IoT random access procedure, which handles different coverage classes, global and local transmission attempts counters, backoff times, and transitions from a class to another, as well as other key simulation. Second, it formulates an analytical model describing both collision and success probabilities, given the number of users willing to perform the random access procedure. Third, it deeply cross-validates the analytical model and the simulation tool, by considering realistic NB-IoT deployments enabling periodic reporting in monitoring infrastructures, with different resource configurations.

**Figure 6:**
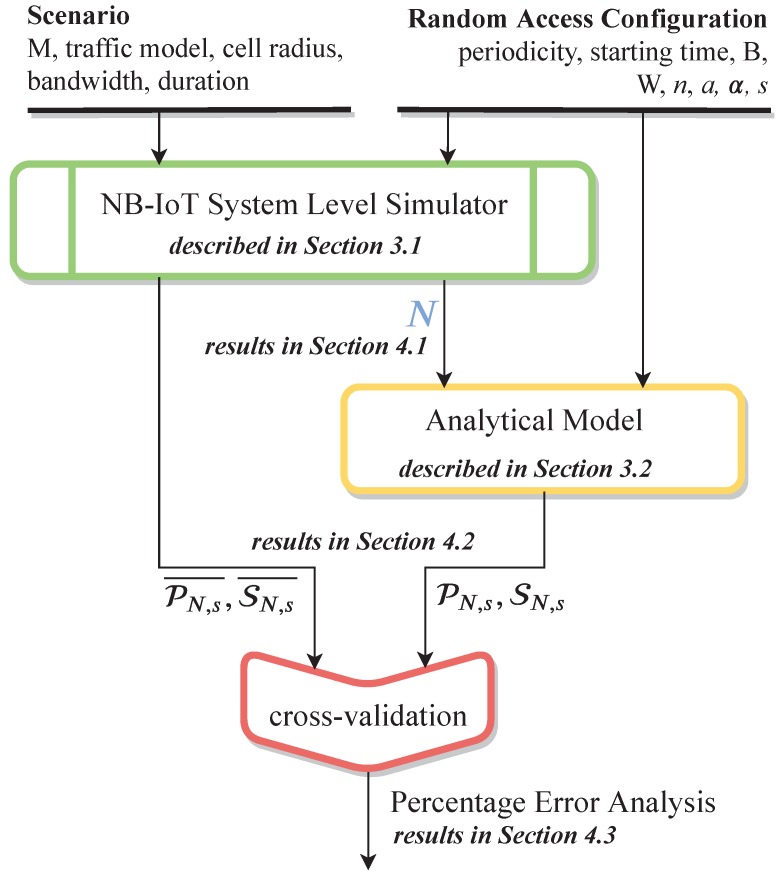
Big picture of the main research contributions.

As outlined in Figure 6, the starting point is the implementation of a 3GPP-compliant NB-IoT random access procedure within an open-source simulation tool [25]. In summary, it is able to provide the number of users accessing the NPRACH and the performance of the procedure itself, expressed in terms of collision probability and success probability, by taking into account a large number of input parameters. Then, the analytical model receives in input the number of users accessing the NPRACH, obtained through computer simulations for estimating both the collision and the success probabilities of users in an RAO.

Note that the symbols adopted in this document are summarized in Table 2.

## 3. The Proposed Analytical Model and Simulation Tool

### 3.1. Implemented Simulation Tool

The proposed tool extends the simulator presented in [25], which is based on the well-known LTE-Sim [48]. The latter is an open-source event-driven simulator which provides a complete performance verification of LTE networks. It was written in C++, using the object-oriented paradigm in order to ensure modularity, polymorphism, flexibility, and high performance. In general, it includes four main components, each of them modeled by a dedicated class: **Simulator** creates, handles and ends events; **NetworkManager** creates and administrates devices; **FlowsManager** manages applications; and **FrameManager** schedules frames and subframes. The parameters related to the spectrum, e.g., frequency carrier, available bandwidth, etc., are handled by the **BandwidthManager** class.

Furthermore, the simulator allows the creation of a specific network scenario as a static function, enabling to model a range of different environments and situations. Every scenario actually creates the aforementioned four basic components, as well as channels, spectrum, cells, devices, Evolved Node-Bs (eNBs), and applications. Then, during the execution of the simulation, the simulator displays several information in real time either on the standard output or on a structured trace file, in order to subsequently and efficiently post-process it.

**Figure 7:**
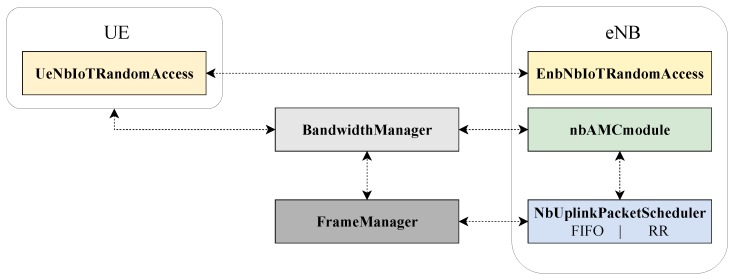
Overview of the main building blocks of the NB-IoT simulation platform.

This simulation platform was later extended for supporting the majority of NB-IoT features. Figure 7 provides an overview of the developed modules for the NB-IoT platform.

Specifically, the **BandwidthManager** class has been drastically changed for handling different subcarrier spacings and *Single-Tone* and *Multi-Tone* transmissions (the latter can be configured for using 3, 6 or 12 tones). Moreover, by modifying the set of frequencies to use, the class natively supports Stand-Alone, In-Band, and Guard-Band operating modes. Multiple carriers can also be enabled by simply defining more channels of 180 kHz each to be used for data transmission. In addition, the **FrameManager** class has been properly updated for controlling the correct length of RUs duration, based on the actual number of tones and the subcarrier spacing chosen for the simulation. Furthermore, a completely redesigned uplink scheduler is realized by means of the **nbUplinkPacketScheduler** class. It provides crucial methods for the management of the RUs. Baseline uplink scheduling strategies, i.e., FIFO and Round-Robin, have been developed as well. A simplified Adaptive Modulation and Coding module (i.e., **nbAMCmodule** class) is used to choose the Transport Block Size (TBS), starting from the selected Modulation and Coding Scheme (MCS) index and number of RUs given by the scheduling strategy, according to 3GPP specification.

**Figure 8:**
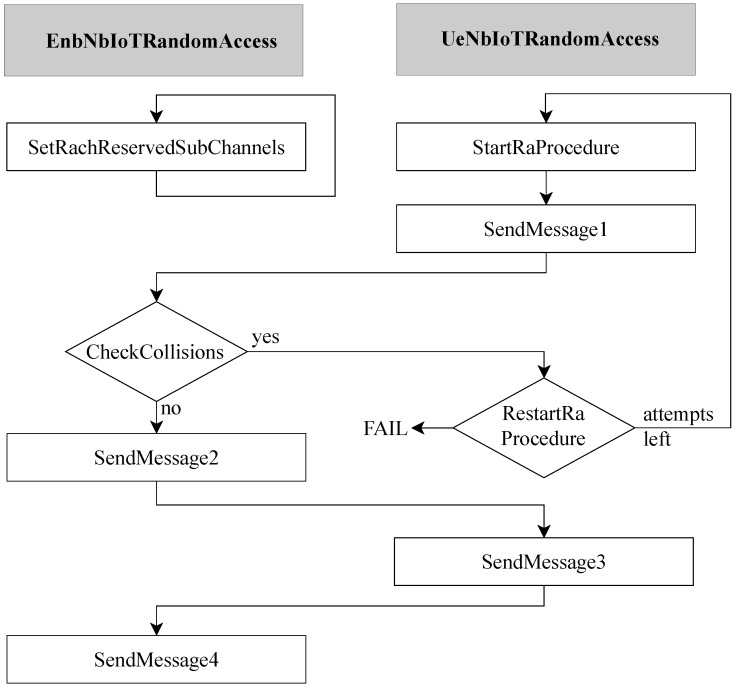
Block diagram of the implemented random access procedure.

Nevertheless, the preliminary version of the NB-IoT simulator did not support the standard-compliant random access procedure. At the code level, the **EnbNbIoTRandomAccess** and **UeNbIoTRandomAccess** classes handle the random access procedure from the base station and user point of view, respectively. Consequently, these classes have been deeply revised since they were not capable of addressing the multi-class attempts control and transitions between coverage classes (as discussed in Section 2.2). In particular, Figure 8 depicts an overview of the implemented code.

With respect to the **UeNbIoTRandomAccess** class, as soon as the traffic generator creates a packet at the application layer, the **UeNbIoTRandomAccess::StartRaProcedure()** method initializes the procedure. Then, several attributes and methods enabling coverage class transitions and attempts-checking are called. In particular, the **UeNbIoTRandomAccess::SendMessage1()** method selects the appropriate NPRACH resources for transmitting preambles in the correct coverage class. In addition, in the **UeNbIoTRandomAccess::ReStartRaProcedure()** method there is an attribute that stores the number of failed attempts for the current coverage class, in order to manage the transitions between coverage classes.

On the other hand, the **EnbNbIoTRandomAccess** class handles the remaining random access procedure features. First, the base station configures NPRACH resources for each coverage class *c*, according to the chosen configuration parameters. Specifically, the base station actually allocates resources to the NPRACH by periodically calling the **EnbNbIoTRandomAccess:: SetRachReservedSubChannels()** method. This means that NPRACH resources must not be used by users for NPUSCH transmission, and, at the same time, they are the only ones where users can send preambles. It is worth mentioning that other configuration parameters, e.g., *B* and *W*, are handled by the base station as well. The **EnbNbIoTRandomAccess::CheckCollisions()** method is of paramount importance. Indeed, for each given RAO of all the coverage class, it checks whether any collisions happened (i.e., two or more users selected the same preamble) and it schedules new preamble transmission after a random backoff time *b*. Then, as explained in Algorithm 1, both attempt counters are incremented and subsequently checked with the chosen values of $a_{c}$ and α, in order to determine a coverage class transition or the procedure failure. Specifically, if the user has any overall attempt left but no more attempts for its class, it switches to the higher class and the number of its attempts for its class is set to zero. If no higher class is available, i.e., the user is already in the *Extreme* class, the user goes on until it has overall attempts left. Next, a new preamble transmission is solicited. If the counter of the overall attempts hits its maximum, the procedure fails and both attempt counters are set to zero. On the contrary, the exchange of the next messages is conducted until the end of the procedure for the users not experiencing a collision. Specifically, the **EnbNbIoTRandomAccess::SendMessage4()** method finalizes the whole random access procedure and makes the end user active and able to transmit data.

Each simulation run provides three main output variables: the number of users accessing an RAO, *N*, as well as the collision and success probabilities, denoted by $\bar{P_{N,s}}$ and $\bar{S_{N,s}}$, respectively.

**Algorithm 1** The implemented random access procedure.
**Require:**
i,j=1
1:get *c*, *n*, *s*, *W*, α, $a_{c}$, *B*2:randomly select one of *s* preambles; wait next RAO; send the preamble3:**if** preamble collided **then**4: i=i+1; j=j+15: draw random *b* from [0,B]6: wait *W*7: **if** j≤α **then**
8:  **if** c=n **then**
9:   wait *b* and go to 210:  **else**
11:   **if** $i\leq a_{c}$ **then**
12:    wait *b* and go to 213:   **else**
14:    i=1; c=c+115:    wait *b* and go to 216:   **end if**
17:  **end if**
18: **else**
19:  procedure FAIL20: **end if**
21:
**else**
22: exchange RAR23: exchange Msg324: exchange Contention Resolution Message25:
**end if**



### 3.2. Formulated Analytical Models

The collision probability and the success probability are analytically derived below, as a function of the actual number of users accessing an RAO, *N*, and the number of available NPRACH subcarriers, *s*. Let the *Collision Probability*, $P_{N,s}$, be the probability that, in a given RAO, a preamble collision happens. First, if no users access the channel, no collisions will occur. Instead, the probability $P_{k}$ that *k* among *N* users choose the same subcarrier follows a binomial distribution. Thus, Pk=Nkpk(1-p)N-k, where *p* is the probability of choosing a specific subcarrier. According to NB-IoT specification, each subcarrier is selected with the same probability, hence $p=\frac{1}{s}$. If a preamble is chosen only once, that is k=1, the probability that only one of the *N* users chooses the specific subcarrier is equal to:

(1) $$P_{1}=N\frac{1}{s}{\left(1-\frac{1}{s}\right)}^{N-1}.$$

Let $\hat{N}$ be the number of users not colliding. It is indeed equal to the average number of preambles chosen only once. In fact, given Equation (1), $\hat{N}$ is computed as the product of $P_{1}$ and the number of different preambles, *s*:

(2) $$\hat{N}=sP_{1}=N{\left(1-\frac{1}{s}\right)}^{N-1}.$$

As a result, the average number of collided users, that is $\tilde{N}$, corresponds to the total amount of users, i.e., *N*, except the users whose preambles not collide, i.e., $\hat{N}$, that is:

(3) $$\begin{array}{c} \tilde{N}=N-\hat{N}=N\left[1-{\left(1-\frac{1}{s}\right)}^{N-1}\right]. \end{array}$$

Thus, the Collision Probability can be expressed as follows:

(4) $$P_{N,s}=\frac{\tilde{N}}{N}=1-{\left(1-\frac{1}{s}\right)}^{N-1},\;N>0.$$

Similarly, let the *Success Probability* be the probability that, in a given RAO, a user successfully completes a preamble transmission. As previously stated, $\hat{N}$ is the number of users not colliding. Since $\hat{N}=N{\left(1-1/s\right)}^{N-1}$, as Equation (2) reports, ${\left(1-1/s\right)}^{N-1}$ clearly represents this success probability. Conversely, the success probability $S_{N,s}$ can be intuitively modeled as:

(5) $$S_{N,s}=1-P_{N,s}={\left(1-\frac{1}{s}\right)}^{N-1},\;N>0.$$

Accordingly, it is worth noting that $S_{1,s}=1,\forall s>0$, as if a single user accesses an RAO, it will certainly not collide.

It is important to emphasize that no stringent assumption on the traffic model has been made in the formulated analytical model. In fact, the probability of a device attempting a preamble transmission in a given RAO depends on both its traffic arrival distribution and NPRACH structure in time. These aspects can be easily managed by the simulation platform.

## 4. Performance Assessment

Cross-validation was conducted to evaluate the accuracy of the implemented simulation platform. According to all the different monitoring systems detailed in Section 2.3, it is reasonable to assume a periodic reporting traffic. Conducted tests consider a 3GPP reference scenario [49], which suitably models highly dense sensor networks. Specifically, transmission requests arrival is modeled according to the uniform distribution in the interval (0,60) s, which is in line with the requirements of a number of smart monitoring applications. In addition, M=5000 or *M* = 10,000 motionless devices are uniformly distributed within a cell with a radius of 1.5 km. Consequently, every second there are, on average, M/60 different mobile terminals that want to transmit a preamble. The base station uses a single NB-IoT carrier of 180 kHz and the uplink is configured in *Single-Tone* mode with a subcarrier spacing of 15 kHz. The chosen uplink scheduling strategy is First In First Out. The simulation environment is summarized in Table 3.

The details related to the considered NPRACH resource configurations are summarized in Table 4 and Table 5.

Note that for Configuration 1 only the *Normal* class is considered, while Configuration 2 contemplates all three coverage classes. It is worth mentioning that, to increase the statistical significance of reported results, each simulation was repeated 150 times with different seeds.

System performance was evaluated in terms of the number of devices involved in the random access procedure, as well as collision and success probabilities. In addition, the analysis of the percentage error between the estimated success probability, i.e., $S_{N,s}$, and the simulated success probability, i.e., $\bar{S_{N,s}}$, was conducted for each set of simulations. The end-to-end delay was statistically analyzed for additional completeness of the performance evaluation. Finally, to provide further performance insights, the impact of RAO periodicity was investigated with a third resource configuration.

### 4.1. Number of Devices Accessing the NPRACH

**Figure 9:**
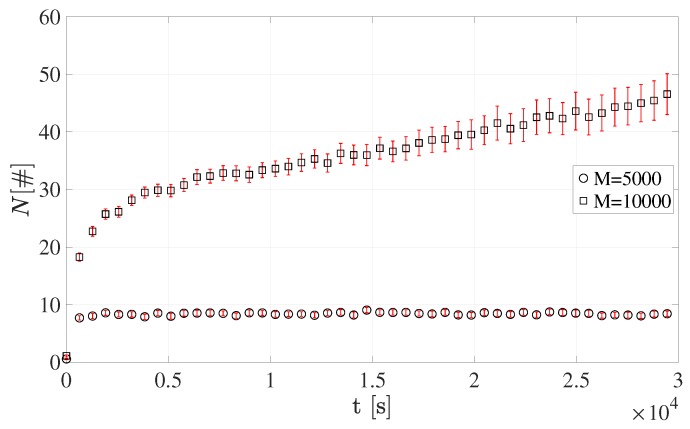
Average number of devices accessing RAOs for Configuration 1.

**Figure 10:**
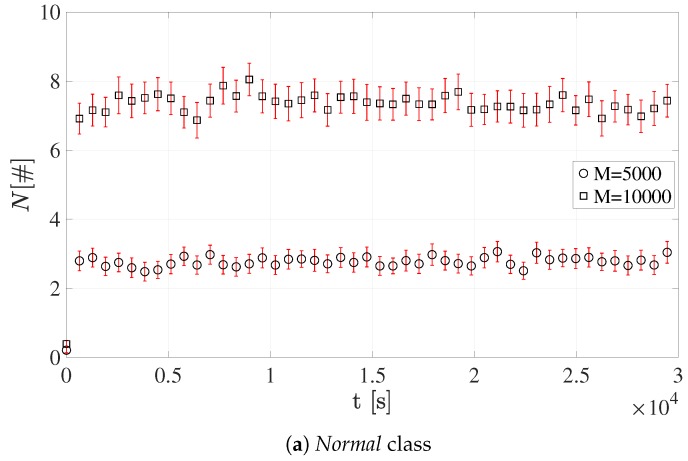

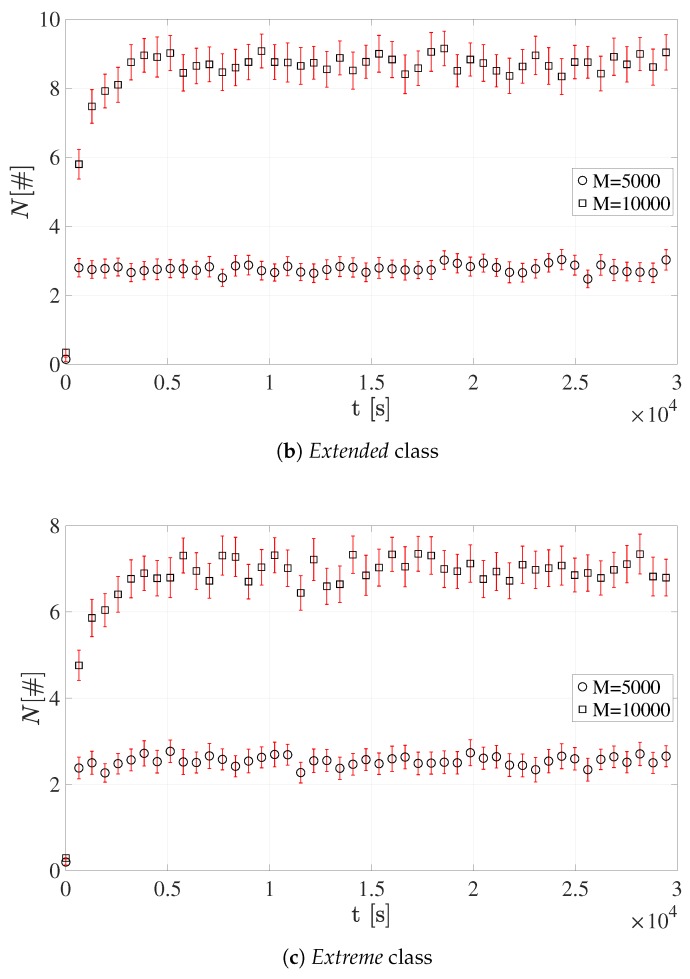
Average number of devices accessing RAOs for Configuration 2.

Figure 9 and Figure 10 show the number of devices accessing RAOs, *N*. It is worth emphasizing that each value is reported together with its 95% confidence interval.

As expected, greater *M* values lead to an overall higher number of terminals in the NPRACH. This evidently holds for all the sets of simulations. The average number of sensors accessing RAOs reaches the highest values in Configuration 1 since all terminals belong to the only available coverage class, i.e., *Normal*. In addition, when *M* = 10,000, the growth of the number of users performing the preamble transmission stems from the scarce amount of NPRACH resources.

Conversely, the average number of devices accessing RAOs is considerably lower in Configuration 2. In fact, mobile terminals are split among the three available coverage classes. It is important to emphasize that *Extended* class presents a higher *N*. This is mainly due both to the low number of different preambles, i.e., s=12, and to the *Normal* class users attempting new transmission in this class. For the same reason, the *Extreme* RAOs should be the most congested; however, the number of different preambles is greater, i.e., s=24.

### 4.2. Collision and Success Probabilities

**Figure 11:**
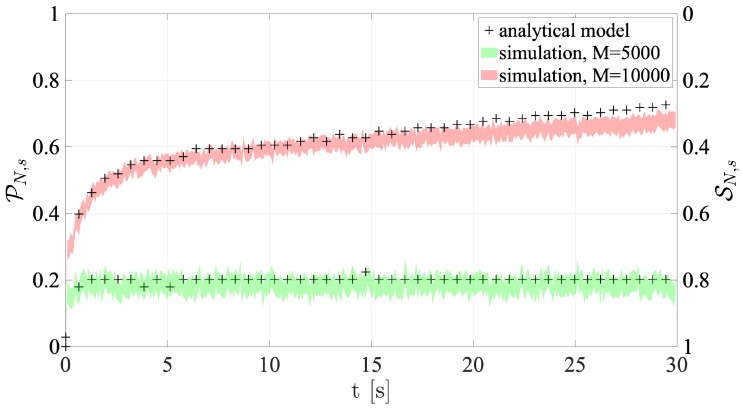
Collision and Success probabilities of RAOs for Configuration 1.

**Figure 12:**
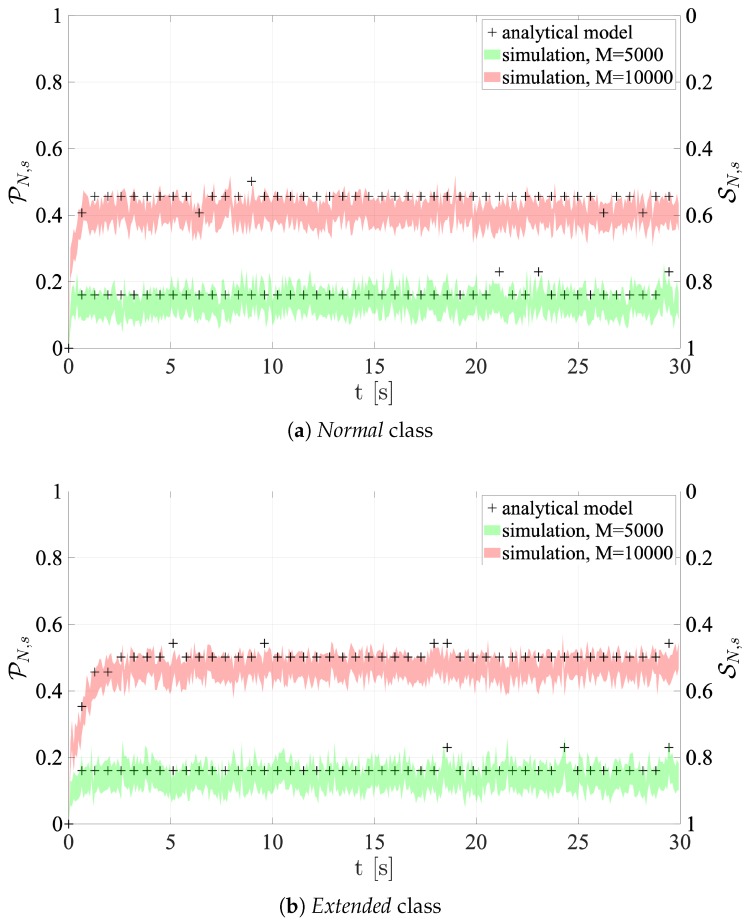

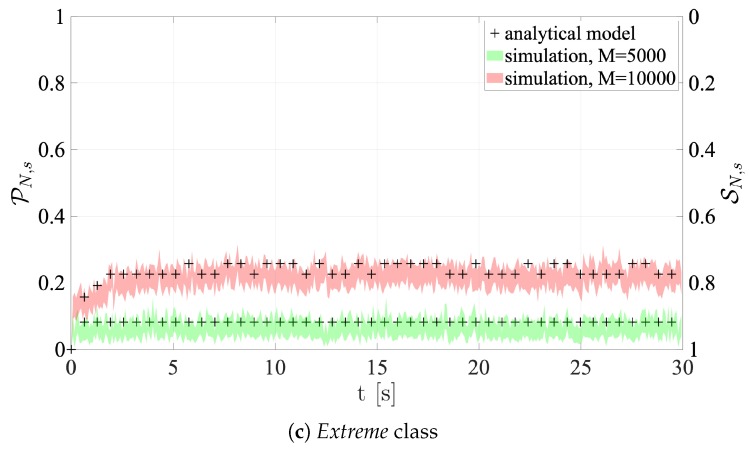
Collision and Success probabilities of RAOs for Configuration 2.

Figure 11 and Figure 12 show the success and collision probabilities in each RAO.

It is important to note that each plot shows both the 95% confidence intervals of the collision and success probabilities directly derived from network simulations, and the probabilities computed using the presented analytical models. In particular, the average number of users accessing the RAOs (which is a value computed by the tool) is utilized as an input for Equations (4) and (5).

The most noticeable feature is that the probabilities obtained by the analytical framework are remarkably consistent with the values given by the proposed simulation platform. Furthermore, the results are also in line with the comments made in the previous section. In fact, when M= 10,000, the collision probability is always higher than the M=5000 case. All figures demonstrate that performance is significantly reduced under high traffic load, especially for Configuration 1. In fact, in accordance with what is mentioned above, Configuration 1 shows the least $S_{N,s}$ values. This depends on the fact that the NPRACH configuration is not adequate to the average number of preamble transmissions. As for Configuration 2, *Extreme* coverage class holds the greatest success probability, since s=24, as outlined above.

### 4.3. Percentage Error of the Success Probabilities

As regards the cross-validation of the formulated model, the discrepancies between the simulated and the analytical success probabilities were quantified in terms of percentage error. This is reported in Table 6 and Table 7, which show its mean value, as well as the 90th and the 10th percentiles. The reported results clearly highlight that the analytical model boasts a good overall accuracy, keeping the mean percentage error always below 6% and the 90th percentile slightly above 10%.

In particular, Configuration 1 outperforms, demonstrating a mean percentage error lower than 3.5%. Nonetheless, in general, the analytical model is more accurate when the first resource configuration is adopted, as also shown in the previous figures. This is mainly due to the fact that in Configuration 2 the average number of devices accessing RAOs is considerably lower. Further analysis could be conducted to demonstrate that the analytical model actually holds higher accuracy when the number of terminals accessing an RAO is relatively large.

### 4.4. End-to-End Delays

**Figure 13:**
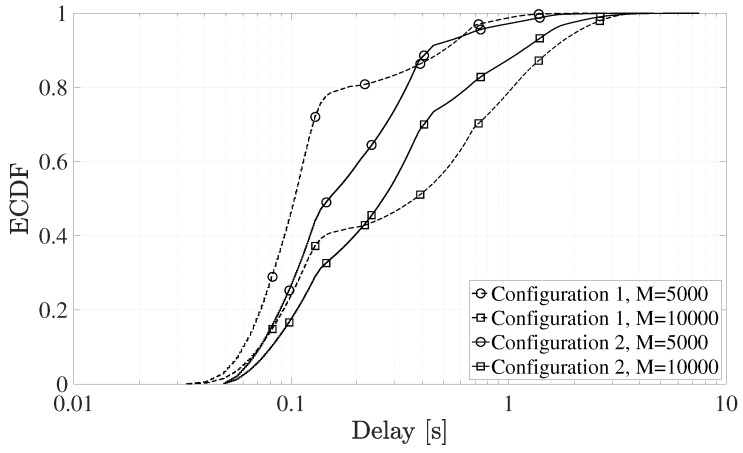
ECDF of the end-to-end delays obtained in all simulations.

Finally, the end-to-end delay was analyzed for all sets of simulations. Figure 13 shows the ECDF of the end-to-end delay experienced by the sensors that successfully complete the random access procedure.

The end-to-end delay is the time interval between the time instant when the devices application layer generates a data packet, and the time instant when that data packet is received by the the base station. Consequently, it includes the access delay as well as the delay introduced by scheduling decisions and the physical transmission of the packet. The most noticeable aspect is that different configurations have different trends. In addition, as expected, the end-to-end delay grows with the number of sensors deployed in the cell, M.

Configuration 1 presents both the best and the worst performance in terms of delay, for M=5000 and M= 10,000, respectively. As a matter of fact, in the first case, there are few user accessing each RAO and a significantly high success probability, as highlighted in the previous sections. Consequently, the end-to-end delay is notably improved, since less preamble retransmission occurs and the uplink scheduler successfully manages the queued terminals. On the contrary, if M= 10,000, the access delay dramatically increase due to the multiple collisions leading to several preamble retransmissions. At the same time, the higher number of successful devices has a negative effect on the waiting time in the scheduling queue as well, hence causing the rise of the end-to-end delay.

Differently, Configuration 2 exhibits an intermediate behavior. This is mainly because longer Backoff times and RAR windows are counterbalanced by the difference in the NPRACH resources structure and higher success probabilities.

### 4.5. Impact of the NPRACH Periodicity

The impact of periodicity is investigated with Configuration 3 since the NPRACH Configurations 1 and 2 do not differ in terms of RAO periodicities. Specifically, the periodicity is doubled compared to the already considered values. The other parameters related to the third NPRACH resource configuration are summarized in Table 8.

**Figure 14:**
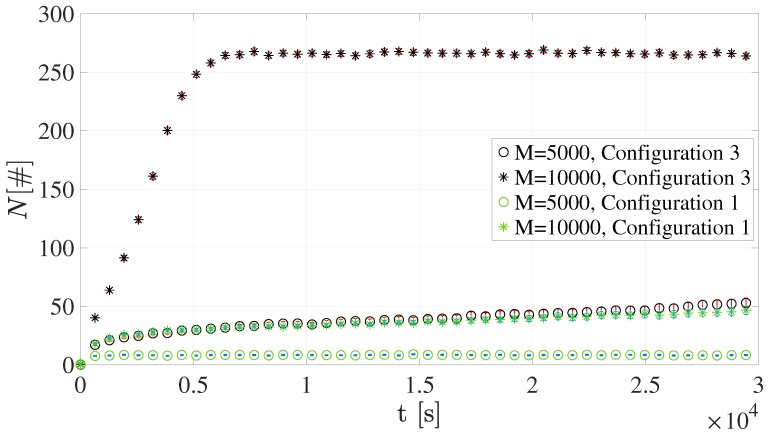
Average number of devices accessing RAOs for Configuration 3.

It is important to note that the only difference with respect to Configuration 1 lies in the periodicity, in order to better identifying its relationship with the number of devices accessing RAOs, as well as the success and collision probabilities. Figure 14 illustrates the number of devices accessing RAOs for both Configuration 3 and Configuration 1.

Greater periodicities values lead to a greater number of terminals in the NPRACH since there are fewer RAOs per unit of time. The average number of sensors accessing RAOs quickly reaches extremely high values when M= 10,000. It is worth noting that the curves do not grow indefinitely because each device can perform the preamble transmission a maximum number of times, i.e., α=10.

**Figure 15:**
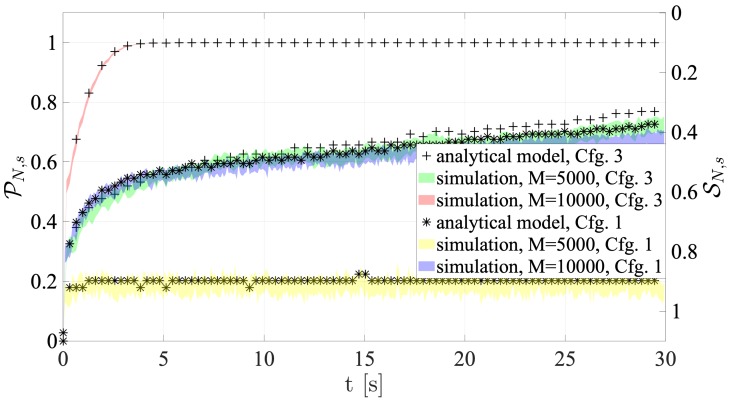
Collision and success probabilities of RAOs for Configuration 3.

In parallel, Figure 15 shows the success and collision probabilities.

The success probability is greatly reduced due to the higher density of terminals in each RAOs. However, it is worth mentioning that, when M=5000, performance is almost satisfactory, however the amount of available resources is halved with respect to Configuration 1.

This brief analysis highlights the important trade-off between the resources available for NPRACH and the resources available for data transmission, i.e., for the NPUSCH.

## 5. Conclusions

NB-IoT is emerging as a promising technology for offering wireless connectivity to constrained smart devices deployed in several pervasive monitoring scenarios, such as Smart City, Precision Agriculture, and Industry 4.0. The random access procedure represents a key relation between devices and the base station, since uplink transmissions prevail among the rest of exchanged messages in a typical NB-IoT monitoring system. Hence, it is of the utmost importance to investigate the behavior of the aforementioned random access procedure.

This paper presents the implementation of an open-source and 3GPP-compliant NB-IoT random access procedure simulation tool and an analytical model describing both collision and success probabilities, given the number of users accessing the random access channel. By taking into account reference applications scenarios based on periodic reporting in monitoring infrastructures, computer simulations showed, as expected, that highly dense sensor networks lead to an overall higher preamble collision probability. Furthermore, cross-validation demonstrated that the formulated analytical model exhibited a considerably low percentage error, proving its remarkable accuracy.

Future activities could involve, on the one hand, the formulation of a complete analytical model to estimate both the number of users performing the random access procedure and an extended analysis of the average end-to-end delay. On the other hand, either entirely new random access mechanisms or major enhancements to the current random access protocol could be implemented in the proposed simulation tool, in order to assess their performance with respect to the standard procedure. Furthermore, the proposed simulation platform could be used for conducting the performance evaluation of different application scenarios, hence embracing the huge extent of Internet of Things use cases.

## Figures and Tables

**Table 1:** Uplink RUs in NB-IoT.

Transmission Mode	Subcarriers	Subcarrier Spacing [kHz]	RU Duration [ms]
Single-Tone	1	3.75	32
	1	15	8
Multi-Tone	3	15	4
	6	15	2
	12	15	1

**Table 2:** Description of adopted symbols.

Symbol	Parameter
*c*	Coverage class
*n*	Number of coverage classes
*s*	Number of NPRACH subcarriers
*W*	RAR Window
α	Maximum transmission attempts
$a_{c}$	Number of transmission attempts in class *c*
*b*	Chosen backoff time
*B*	Maximum backoff time
*N*	Number of users accessing an RAO
*M*	Number of users in the cell
$P_{N,s}$	Collision Probability
$\bar{P_{N,s}}$	Simulated Collision Probability
$S_{N,s}$	Success Probability
$\bar{S_{N,s}}$	Simulated Success Probability

**Table 3:** Summary of the simulation environment.

Parameter	Value
Traffic model	Uniform Distribution—(0, 60) s
Number of sensors (*M*) [#]	5000, 10,000
Sensors spatial distribution	Uniform
Cell radius [km]	1.5
NB-IoT bandwidth [kHz]	180
Transmission mode	Single-Tone
Subcarrier spacing [kHz]	15
Uplink scheduler	FIFO

**Table 4:** NPRACH Configuration 1 [50].

Parameter	Value
α [#]	10
*n* [#]	1
*c*	*Normal*
z$a_{c}$ [#]	6
α [#]	10
periodicity [ms]	80
*s* [#]	36
*B* [ms]	512
*W* [ms]	24

**Table 5:** NPRACH Configuration 2 [50].

Parameter	Value		
α [#]	10		
*n* [#]	3		
*c*	*Normal*	*Extended*	*Extreme*
$a_{c}$ [#]	3	3	6
periodicity [ms]	80	80	80
*s* [#]	12	12	24
*B* [ms]	256	512	1024
*W* [ms]	12	48	256

**Table 6:** Percentage error between success probabilities for Configuration 1.

*M* [#]	Mean	90th Percentile	10th Percentile
5000	1.7	3.2	0.3
10,000	3.4	6.5	0.6

**Table 7:** Percentage error between success probabilities for Configuration 2.

Class	*M* [#]	Mean	90th Percentile	10th Percentile
*Normal*	5000	6.0	10.4	1.6
*Extended*	5000	5.9	10.5	1.4
*Extreme*	5000	2.5	10.5	1.4
*Normal*	10,000	3.4	7.1	0.4
*Extended*	10,000	4.2	8.1	0.7
*Extreme*	10,000	2.6	8.1	0.4

**Table 8:** NPRACH Configuration 3.

Parameter	Value
α [#]	10
*n* [#]	1
*c*	***Normal***
$a_{c}$ [#]	6
α [#]	10
periodicity [ms]	160
*s* [#]	36
*B* [ms]	512
*W* [ms]	24
